# Proteinuria Is Associated with Carotid Artery Atherosclerosis in Non-Albuminuric Type 2 Diabetes: A Cross-Sectional Study

**DOI:** 10.3390/jcm9010136

**Published:** 2020-01-03

**Authors:** Jaehyun Bae, Yong-ho Lee, Eun Seok Kang, Bong-Soo Cha, Byung-Wan Lee

**Affiliations:** Division of Endocrinology and Metabolism, Department of Internal Medicine, Yonsei University College of Medicine, Seoul 03722, Korea; janggu1985@yuhs.ac (J.B.); YHOLEE@yuhs.ac (Y.-h.L.); edgo@yuhs.ac (E.S.K.); bscha@yuhs.ac (B.-S.C.)

**Keywords:** non-albuminuric proteinuria, type 2 diabetes, atherosclerosis

## Abstract

The association of specific urinary proteins other than albumin with cardiovascular (CV) outcomes in patients with type 2 diabetes (T2D) has been shown. In this respect, CV outcomes may differ in non-albuminuric T2D patients who were considered as a low risk group, according to the presence of proteinuria. We investigated the association between proteinuria and atherosclerosis assessed by carotid artery intima-media thickness (CIMT) in non-albuminuric T2D patients. 2047 T2D patients whose urine albumin-to-creatinine ratio was below 30 mg/g were recruited and classified into a non-proteinuria (NP, uPCR < 150 mg/g, n = 1865) group and a non-albuminuric proteinuria (NAP, uPCR ≥ 150 mg/g, n = 182) group. CIMT was compared between the two groups and logistic regression analysis was conducted to verify whether proteinuria could predict deteriorated CIMT status. In this cross-sectional study, mean CIMT of the NAP group were significantly thicker than those of the NP group (0.73 ± 0.16 vs. 0.70 ± 0.14, p = 0.016). The presence of proteinuria is associated with deteriorated CIMT after the adjustment for conventional risk factors (odds ratio, 2.342; 95% confidence interval, 1.082–5.070, p = 0.030) in regression analysis. We postulated that the measurement of urinary protein in conjunction with albumin might be helpful for predicting atherosclerosis, especially for non-albuminuric patients.

## 1. Introduction

Cardiovascular disease (CVD), the main pathological consequence of atherosclerosis, is a major factor of morbidity and mortality in patients with type 2 diabetes (T2D) [1,2]. Therefore, the early detection of atherosclerosis to screen for CVD in T2D patients is urgently needed. Several surrogate markers for subclinical atherosclerosis have been introduced, such as carotid artery intima-media thickness (CIMT), ankle-brachial index (ABI), and pulse wave velocity (PWV) [3,4,5]. 

CIMT measured by carotid ultrasonography is one of the most frequently performed tests for screening T2D patients for atherosclerosis. Because of a lack of a uniform methodology, it remains controversial whether CIMT could be a consistent screening tool for atherosclerosis. However, CIMT is a clinically important screening tool for atherosclerosis because it is convenient to perform and there is abundant evidence of its association with CVD or vascular complications of T2D [6,7,8,9].

Along with the estimated glomerular filtration rate (eGFR), the quantification of urinary albumin is generally recommended to screen for diabetic kidney disease (DKD) in T2D patients [10]. Increased albuminuria is defined as ≥30 mg/g creatinine, and this abnormal condition has been reported to be associated with CVD and mortality [11,12,13,14] as well as glomerular damage [15]. However, it is well known that DKD may progress without increases in albuminuria [16,17].

In this context, several tubular damage markers such as fatty acid-binding proteins (FABPs) and *N*-acetyl-β-d-glucosaminidase (NAG) have gained attention for their correlations with DKD [18,19]. These markers, specific urinary proteins other than albumin, are also associated with other diabetic complications and CVD [20,21,22,23,24,25,26]. These previous studies suggest that even in non-albuminuric T2D patients, there was a significant difference in the risk or status of vascular complications depending on the presence of proteinuria. Therefore, in this study, we recruited T2D patients without albuminuria and evaluated the association between total proteinuria and atherosclerosis assessed by CIMT.

## 2. Materials and Methods

### 2.1. Study Population

This retrospective cross-sectional observational study included patients who visited the diabetes centre at Severance Hospital in Seoul, South Korea, between July 2015 and July 2018. A total of 2047 non-albuminuric patients with T2D who simultaneously underwent laboratory measurements and carotid ultrasonography were recruited. A non-albuminuric patient was defined as having a urine albumin-to-creatinine ratio (ACR) of <30 mg/g. T2D was defined as: (1) use of insulin or antihyperglycemic agents (AHAs) or (2) a glycated haemoglobin (HbA1c) level ≥ 6.5%. Patients were excluded if they fulfilled any one of the following criteria: (1) <19 years of age; (2) current pregnancy; (3) end stage renal disease (defined as eGFR < 15 mL/min/1.73 m^2^); and (4) presence of significantly elevated transaminases (aspartate aminotransferase or alanine aminotransferase ≥5 times the upper limit of normal).

Demographic data such as age, sex, blood pressure, and body mass index (BMI) were retrospectively collected from the patients’ electronic medical records. BMI was calculated as weight divided by height squared (kg/m^2^). We collected records of the medications related to atherosclerosis such as anti-platelet agents and lipid-lowering agents by searching the electronic medical records. We also identified medical history of hypertension (HTN) by antihypertensive medication usage and International Classification of Disease 10th revision (ICD-10) diagnosis codes as followed; I10.0, I10.1, I10.9. Finally, to assess the vascular condition of subjects, we reviewed ICD-10 codes of cardiovascular diseases (CVDs) as followed; I20.x, I21.x, I22.x, I24.x, I25.x, I60.x, I61.x, I63.x–I69.x, G45.x (not G45.4).

The study protocol received ethical approval from the institutional review board at the Yonsei University College of Medicine (No. 4-2019-0107), which waived the need for informed consent because the database was only retrospectively accessed for analytical purposes and personal information was not used.

### 2.2. Measurements of Clinical and Laboratory Parameters

Following an overnight fast (≥8 h), blood samples were obtained to measure glucose, HbA1c, lipid profile, blood urea nitrogen (BUN), creatinine, and liver enzymes. Low-density lipoprotein cholesterol (LDL-C) was calculated using the Friedewald equation if there was no actual LDL-C measurement [27]. If a patient’s triglyceride (TG) level exceeded 400 mg/dL, we did not use the equation. Spot urine tests for proteinuria, including protein, albumin, and creatinine, were performed using an AU680 analyzer (Beckman Coulter Inc., Brea, CA, USA). Calculated urine ACR was used as inclusion or exclusion criteria. Proteinuria was defined as urine protein-to-creatinine ratio (PCR) ≥ 150 mg/g [28]. The eGFR was calculated using the Modification of Diet in Renal Disease study equation [29].

### 2.3. Measurements of CIMT

Two specialized technicians conducted common carotid arterial ultrasound examinations using an Aplio 500 instrument (Toshiba, Tokyo, Japan) equipped with a 13-MHz linear probe using CIMT measurement protocols described in detail previously [30]. In brief, CIMT were recorded with the subject in a supine position with the head elevated to 45° and tilted to either side by 30°. We performed B mode examinations about 1.5 cm proximal to the carotid bifurcation on the far wall of the common carotid artery and on both sides. IMT was defined as the distance between the media–adventitia interface and the lumen–intima interface. Mean CIMT was defined as the mean IMT of the right and left carotid arteries. Mean of maximum CIMT was defined as the mean maximum IMT of the right and left carotid arteries. Carotid plaques were determined according to Mannheim consensus [31]. The presence of carotid plaques was positive when one or more carotid plaques existed. Considering previous studies and recommendations, we defined an abnormal (or significantly increased) CIMT as an absolute mean IMT value of ≥ 1 mm [6,32].

### 2.4. Statistical Analysis

The baseline characteristics, laboratory measurements, and CIMT were compared according to proteinuria status. Student’s t-test and the *X*^2^ test were used to compare continuous and categorical variables between the two groups. Correlations between proteinuria, CIMT, and other independent variables were analysed with Spearman’s correlation coefficients. 

Multiple logistic regression analyses were performed to evaluate whether proteinuria could predict an abnormal CIMT. First, we conducted the unadjusted logistic regression analysis. And then, we made three multiple logistic regression models adjusted by various factors which could affect CIMT, such as age, sex, history of CVD and blood cholesterol level. The logistic regression analysis results are presented as odds ratio (OR) and 95% confidence interval (CI). To assess the predictive accuracy of proteinuria adjusted by multiple variables, we derived receiver operating characteristics curves of the logistic regression models for abnormal CIMT and calculated areas under the curve (AUC). 

All statistical analyses were performed using SPSS version 21.0 for Windows (IBM Corp., Armonk, NY, USA). Continuous variables are expressed as mean ± standard deviation (SD), while categorical variables are shown as number and percentage (%). p Values < 0.05 were considered statistically significant.

## 3. Results

### 3.1. Study Population Characteristics

Of the 2047 non-albuminuric T2D patients, 1865 were non-proteinuric (NP) and the remaining 182 had proteinuria (non-albuminuric proteinuria [NAP]) (Table 1). The mean urine PCR was 87.6 mg/g in the NP group and 205.6 mg/g in the NAP group.

Patients in the NAP group tended to be leaner (mean BMI, 24.4 ± 3.3 vs. 25.5 ± 3.5, p = 0.002) and older (64.4 ± 9.6 vs. 59.7 ± 10.7, p < 0.001). NAP patients also showed lower diastolic blood pressure (71.6 ± 10.7 vs. 74.2 ± 10.3, p = 0.009). The percentage of patients with CVDs was higher (47.3% vs. 39.7%, p = 0.047), and drinking history was lower (12 of 49 vs 297 of 581 patients who had related records, p < 0.001) in NAP group. Serum glucose and HbA1c were significantly higher in the NAP group (142.8 ± 47.6 vs. 133.3 ± 38.3, p = 0.009; 7.6 ± 1.6 vs. 7.1 ± 1.2, p < 0.001). Blood lipid profiles were generally lower in NAP; total cholesterol (154.5 ± 32.3 vs. 159.8 ± 33.7, p = 0.043), high-density lipoprotein cholesterol (HDL-C, 46.0 ± 11.9 vs. 47.8 ± 11.2, p = 0.038), and LDL-C (80.2 ± 29.5 vs. 86.3 ± 30.1, p = 0.009) were higher in the NP group. 

BUN levels of the NAP patients were higher than those of the NP group (17.2 ± 5.6 vs. 15.6 ± 4.6, p < 0.001), but there was no significant intergroup difference in renal function (eGFR, 89.7 ± 27.7 vs. 93.3 ± 22.6, p = 0.093) and no significant intergroup differences in liver enzymes or history of antiplatelet or lipid lowering agent usage.

Mean CIMT and mean of maximum CIMT were significantly increased in the NAP group (0.73 ± 0.16 vs. 0.70 ± 0.14, p = 0.016; 0.86 ± 0.21 vs. 0.82 ± 0.17, p = 0.008). Patients in the NAP group also tended to have carotid plaques (136 of 182 (74.7%) vs. 1259 of 1865 (67.5%), p = 0.046).

### 3.2. Variables Correlated with Significantly Increased CIMT

In Spearman’s correlation analysis, mean CIMT was positively correlated with age, CVD history, urine PCR, and serum creatinine but negatively correlated with female, diastolic blood pressure, eGFR, total cholesterol, TG, and HDL-C (Table 2). BMI, smoking or drinking history, and parameters related to glycaemic control such as fasting glucose and HbA1c were not significantly correlated with CIMT.

### 3.3. OR for Significantly Increased CIMT with Proteinuria

We performed multiple logistic regression analyses to investigate the association between the presence of proteinuria (PCR ≥ 150 mg/g) and a significantly increased mean CIMT (≥1 mm). 

**Figure 1:**
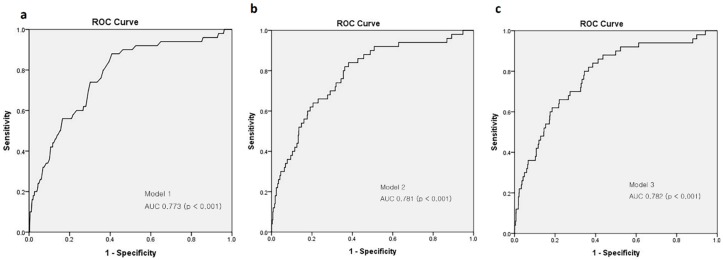
Receiver Operating Characteristic (ROC) curves for the multivariate logistic regression models. Model 1 (**a**) adjusted for age, sex, presence of proteinuria, model 2 (**b**) further adjustment for HbA1c and total cholesterol to model 1, model 3 (**c**) further adjustment for diastolic blood pressure, history of CVD, eGFR, and lipid lowering medication to model 2.

In an unadjusted model, the presence of proteinuria was considerably associated with an abnormal CIMT (OR, 3.8; 95% CI, 1.981–7.289; p < 0.001) (Table 3). When we built a model adjusted for age and sex (model 1), the presence of proteinuria was still substantially associated with an abnormally increased CIMT (OR, 2.765; 95% CI, 1.413–5.411; p = 0.003). After further adjustment for HbA1c and total cholesterol to model 1 (model 2), proteinuria remained statistically significant (OR, 2.395; 95% CI, 1.206–4.754; p = 0.013). Finally, we additionally adjusted diastolic blood pressure, history of CVD, eGFR, and lipid lowering agent to model 2 (model 3) and found that the presence of proteinuria was still significantly associated with abnormal CIMT (OR, 2.881; 95% CI, 1.329–6.244; p = 0.007). Each model showed meaningful predictive power (AUC of model 1, 0.773; model 2, 0.776, model 3, 0.771; *p* value of all models < 0.001) (Figure 1).

## 4. Discussion

In this study, we found that overt proteinuria is significantly associated with deteriorated CIMT in non-albuminuric T2D patients. Proteinuria maintained its predictive power for an increased CIMT in non-albuminuric patients after the adjustment for age, sex, diastolic blood pressure, history of CVD, HbA1c, eGFR, total cholesterol, and lipid lowering medication. These results indicate that, even in patients without albuminuria, the risk or status of vascular complications may differ in the presence of proteinuria.

Albuminuria and eGFR are the currently established tools for DKD screening in T2D patients [10,33]. Albuminuria, usually defined as ≥30 mg/g creatinine, has shown its association with renal outcome [34,35], cardiovascular disease [13,36], and mortality [11,12]. Albuminuria is also recognized as a more sensitive marker than eGFR. However, in some cases, DKD progresses without significant albuminuria [16,17] since it is detected only after glomerular damage occurs. The pathophysiology of DKD is complex; various pathways and factors, such as the renin-angiotensin-aldosterone system, inflammatory responses, oxidative stress, and renal hemodynamic changes, are involved in DKD [37]. Therefore, albuminuria, a marker of glomerular damage, might be insufficient to reflect early-stage DKD, which encompasses a wide range of mechanisms. In particular, albuminuria is not sensitive to tubular damage, which is also an important component of early DKD pathology [38,39]. Because the vascular complications of T2D share pathophysiologic mechanisms, this limitation of albuminuria highlights that vascular complications of T2D such as CVD may differ, even in patients without albuminuria. 

Several urinary proteins that reflect tubular damage, such as FABPs, NAG, neutrophil gelatinase-associated lipocalin (NGAL), and kidney injury molecule-1 (KIM-1) were introduced as complimentary markers to albuminuria for DKD. Although there were some conflicting results, these biomarkers also have shown their associations with CVD; urinary liver-type FABP showed correlations with the development of end stage renal disease and CVD [40]. The association between NAG and CVD was reported mainly in diabetic patients [20,22]. In addition, NGAL and KIM-1 have shown their predictive value for CVD outcomes in various studies [24,25,41]. In particular, KIM-1 was independently associated with atherosclerotic CVD [23].

However, among the abovementioned biomarkers, no single marker has shown sufficient evidence or clinical cost-effectiveness to be introduced as a standard screening strategy like albuminuria. Moreover, assessing multiple biomarkers is inefficient and impractical. Therefore, total proteinuria, which includes these tubular proteins, is a useful, practical screening tool, especially for non-albuminuric T2D patients. Assessing total urinary protein or identifying NAP patients would be meaningful in this context, especially for elderly T2D patients whose progression rate to CKD is usually high [42].

Previous studies have shown that NAP predicts DKD progression [43,44]. Our research team also reported that patients with NAP were more closely associated with decreased beta cell function and an increased prevalence of vascular disease [45]. However, few studies have focused on the relationship between NAP and markers of subclinical atherosclerosis. Moreover, to our knowledge, no study has evaluated the associations between total proteinuria and atherosclerosis in non-albuminuric T2D patients. In this study, we recruited non-albuminuric T2D patients and demonstrated that NAP patients show a higher prevalence of deteriorated atherosclerosis assessed by CIMT than NP patients.

We acknowledge that this study has several limitations. First, owing to its retrospective cross-sectional study design, we could not make any inference of causality. Second, the numerical imbalance due to the relatively few patients in the NAP group could be a limitation of the lack of statistical power. We tried to minimize that limitation by recruiting as large a population as possible (over 2000 patients). Third, the comparison of CIMT between NAP group and albuminuria group was not conducted. Therefore, we could not demonstrate CIMT status in the NAP group or the predictive power of NAP for deteriorated CIMT versus that in albuminuric patients. However, we think that demonstrating the need for total proteinuria quantification in conjunction with albuminuria to predict atherosclerosis, especially in non-albuminuric patients, has sufficient clinical significance. Further studies comparing the NAP and albuminuria groups in terms of carotid artery atherosclerosis (CAA) are needed. Fourth, because this study included patients who visited the diabetes centre of Severance Hospital, we could not recruit non-diabetic subjects, so our findings cannot be applied to non-diabetic patients. Fifth, the list of proteinuria, which would be composed of albumin and various tubular injury markers, was not examined in this study because we retrospectively used routine laboratory measurements. Further studies which assess the composition of urinary protein in NAP group are needed. Sixth, since there were two technicians for CIMT measurement, inter-observer variation may have occurred. Finally, the cut-off value for abnormal CIMT in this study, 1 mm, may be controversial because there is no established single reference value for progressed CIMT. Although the American Society of Echocardiography Consensus Statement recommended that CIMT ≥ 75th percentile for age, sex, and ethnicity indicates high risk [32], there is a lack of CIMT percentile data by age and sex for Koreans. In addition, since this study excluded albuminuric patients, the use of in-group percentile as a reference point is inappropriate because it could not reflect the absolute high-risk status. Considering this context, we sought to find the absolute reference value of CIMT and determined the cut-off value for deteriorated CIMT as 1 mm on the basis of abundant previous studies that proved its clinical implications or used it as a cut-off point [6,46,47,48,49].

## 5. Conclusions

In this study, we showed that the presence of overt proteinuria is significantly associated with deteriorated status of CAA in non-albuminuric T2D patients. This result suggests that the measurement of urinary protein in conjunction with albumin might be helpful for predicting CAA. 

## Figures and Tables

**Table 1:** Participants’ demographic and laboratory characteristics.

Characteristic (unit)	NP (n = 1865)	NAP (n = 182)	p Value
Age (years)	59.7 ± 10.7	64.4 ± 9.6	<0.001
Male, n (%)	1049 (56.2%)	98 (53.8%)	0.533
BMI (kg/m^2^)	25.5 ± 3.5	24.4 ± 3.3	0.002
Systolic blood pressure (mmHg)	123.8 ± 12.0	122.3 ± 13.8	0.189
Diastolic blood pressure (mmHg)	74.2 ± 10.3	71.6 ± 10.7	0.009
History of HTN, n (%)	952 (51.0%)	100 (54.9%)	0.369
History of CVD, n (%)	740 (39.7%)	86 (47.3%)	0.047
Coronary heart disease, n (%)	333 (17.9%)	41 (22.5%)	0.119
Other cerebrovascular disease, n (%)	554 (29.7%)	63 (34.6%)	0.168
Smoking history (non/ex-/current), n *	317/159/122	35/8/8	0.090
Drinking history (no/yes), n *	284/297	37/12	<0.001
HbA1c (%)	7.1 ± 1.2	7.6 ± 1.6	<0.001
Fasting glucose (mg/dL)	133.3 ± 38.3	142.8 ± 47.6	0.009
AST (IU/L)	23.6 ± 11.5	23.8 ± 16.6	0.882
ALT (IU/L)	25.2 ± 16.1	25.9 ± 21.9	0.649
Total bilirubin (mg/dL)	0.8 ± 0.3	0.7 ± 0.4	0.044
BUN (mg/dL)	15.6 ± 4.6	17.2 ± 5.6	<0.001
Creatinine (mg/dL)	0.8 ± 0.2	0.8 ± 0.3	0.065
eGFR (MDRD, mL/min/1.73 m^2^)	93.3 ± 22.6	89.7 ± 27.7	0.093
Total cholesterol (mg/dL)	159.8 ± 33.7	154.5 ± 32.3	0.043
Triglyceride (mg/dL)	130.1 ± 64.2	137.1 ± 72.7	0.165
HDL-C (mg/dL)	47.8 ± 11.2	46.0 ± 11.9	0.038
LDL-C (mg/dL)	86.3 ± 30.1	80.2 ± 29.5	0.009
Urine PCR (mg/g creatinine)	87.6 ± 25.5	205.6 ± 72.5	<0.001
Mean CIMT (mm)	0.70 ± 0.14	0.73 ± 0.16	0.016
Mean of maximum CIMT (mm)	0.82 ± 0.17	0.86 ± 0.21	0.008
Presence of carotid plaques, n (%)	1259 (67.5%)	136 (74.7%)	0.046
Usage of antiplatelet agent, n (%)	672 (36.0%)	75 (41.2%)	0.166
Usage of lipid lowering agent, n (%)	940 (50.4%)	88 (48.4%)	0.597

Continuous variables are expressed as mean ± standard deviation (SD). * Due to relatively small number of evaluable patient, only number of subjects were presented, without percentage of each group. NP, non-proteinuric group; NAP, non-albuminuric proteinuria group; BMI, body mass index; HTN, hypertension; CVD, cardiovascular disease; HbA1c, glycated haemoglobin; AST, aspartate aminotransferase; ALT, alanine aminotransferase; BUN, blood urea nitrogen; eGFR, estimated glomerular filtration rate; MDRD, Modification of Diet in Renal Disease study equation; HDL-C, high-density lipoprotein cholesterol; LDL-C, low-density lipoprotein cholesterol; PCR, protein-to-creatinine ratio; CIMT, carotid artery intima-media thickness.

**Table 2:** Correlations between mean CIMT and other variables.

Characteristic (unit)	All Patients (N = 2047)	
	*r*	p Value
Age (years)	**0.525**	**<0.001**
Sex (Female vs. male)	**−0.111**	**<0.001**
BMI (kg/m^2^)	−0.027	0.342
Systolic blood pressure (mmHg)	0.024	0.372
Diastolic blood pressure (mmHg)	−0.193	<0.001
History of CVD	0.224	<0.001
Smoking history	−0.006	0.870
Drinking history	0.047	0.243
Urine PCR (mg/g creatinine)	**0.074**	**<0.001**
HbA1c (%)	−0.043	0.051
Fasting Glucose (mg/dL)	−0.011	0.633
Creatinine (mg/dL)	**0.157**	**<0.001**
eGFR (MDRD, mL/min/1.73 m^2^)	**−0.149**	**0.041**
Total cholesterol (mg/dL)	**−0.098**	**<0.001**
Triglyceride (mg/dL)	**−0.046**	**0.037**
HDL-C (mg/dL)	**−0.102**	**<0.001**
LDL-C (mg/dL)	−0.040	0.067

BMI, body mass index; CVD, cardiovascular disease; PCR, protein-to-creatinine ratio; HbA1c, glycated haemoglobin; eGFR, estimated glomerular filtration rate; MDRD, Modification of Diet in Renal Disease Study equation; HDL-C, high-density lipoprotein cholesterol; LDL-C, low-density lipoprotein cholesterol; CIMT, carotid artery intima-media thickness.

**Table 3:** Odds ratios for significantly increased CIMT by proteinuria.

Variables	OR	95% CI	p-Value
**Unadjusted**			
Presence of proteinuria	3.800	1.981–7.289	<0.001
**Model 1**			
Age (years)	1.099	1.062–1.138	<0.001
Sex (female vs. male)	0.584	0.322–1.059	0.077
Presence of proteinuria	2.765	1.413–5.411	0.003
**Model 2**			
Age (years)	1.100	1.063–1.139	<0.001
Sex (female vs. male)	0.608	0.333–1.110	0.105
HbA1c (%)	1.249	1.039–1.501	0.018
Total cholesterol (mg/dL)	0.994	0.985–1.004	0.236
Presence of proteinuria	2.395	1.206–4.754	0.013
**Model 3**			
Age (years)	1.107	1.062–1.153	<0.001
Sex (female vs. male)	0.677	0.333–1.378	0.282
Diastolic blood pressure (mmHg)	0.974	0.942–1.008	0.134
History of CVD	1.056	0.511–2.183	0.882
HbA1c (%)	1.285	0.999–1.653	0.051
eGFR (MDRD, mL/min/1.73 m^2^)	1.001	0.987–1.015	0.872
Total cholesterol (mg/dL)	0.993	0.981–1.004	0.209
Usage of lipid lowering agent	0.835	0.405–1.721	0.625
Presence of proteinuria	2.881	1.329–6.244	0.007

OR, odds ratio; CI, confidence interval; HbA1c, glycated haemoglobin; eGFR, estimated glomerular filtration rate; MDRD, Modification of Diet in Renal Disease Study equation; CIMT, carotid artery intima-media thickness.
